# *In vitro* Activity of a New Fourth-Generation Cephalosporin, Cefoselis, Against Clinically Important Bacterial Pathogens in China

**DOI:** 10.3389/fmicb.2020.00180

**Published:** 2020-02-28

**Authors:** Jing-Wei Cheng, Jian-Rong Su, Meng Xiao, Shu-Ying Yu, Ge Zhang, Jing-Jia Zhang, Yang Yang, Si-Meng Duan, Timothy Kudinha, Qi-Wen Yang, Ying-Chun Xu

**Affiliations:** ^1^Department of Clinical Laboratory, Peking Union Medical College Hospital, Peking Union Medical College, Chinese Academy of Medical Sciences, Beijing, China; ^2^Center of Clinical Laboratory, Beijing Friendship Hospital, Capital Medical University, Beijing, China; ^3^Beijing Key Laboratory for Mechanisms Research and Precision Diagnosis of Invasive Fungal Diseases (BZ0447), Beijing, China; ^4^Graduate School, Peking Union Medical College, Chinese Academy of Medical Sciences, Beijing, China; ^5^School of Biomedical Science, Charles Sturt University, Orange, NSW, Australia; ^6^Centre for Infectious Diseases and Microbiology Laboratory Services, ICPMR – Pathology West, Westmead Hospital, The University of Sydney, Sydney, NSW, Australia

**Keywords:** cefoselis, cefepime, antimicrobial resistance, *in vitro* activity, China

## Abstract

The objective of this study was to systematically evaluate the *in vitro* activity of cefoselis and other comparators against common bacterial pathogens collected from 18 hospitals across China. Minimum inhibitory concentrations (MICs) were determined by the broth microdilution method following Clinical and Laboratory Standards Institute (CLSI) guidelines. Cefoselis showed poor activity against extended-spectrum β-lactamase (ESBL)-producing *Escherichia coli*, *Klebsiella pneumoniae*, and *Proteus mirabilis*, with susceptibility rates of < 10% each, while the susceptibility rates of this antibiotic against non-ESBL-producing strains of these organisms were 100%, 94.3%, and 97.0%, respectively. Cefoselis exhibited susceptibility rates of 56.7–83.3% against other tested Enterobacteriaceae isolates. For *Acinetobacter baumannii* and *Pseudomonas aeruginosa* isolates, the susceptibility rates to cefoselis were 18.7% and 73.3%, respectively. All methicillin-resistant *Staphylococcus aureus* (MRSA) strains were resistant to cefoselis, while all methicillin-sensitive *S. aureus* (MSSA) strains were susceptible to this antibiotic. In conclusion, cefoselis showed good activity against non-ESBL-producing *E. coli*, *K. pneumoniae*, and *P. mirabilis*, MSSA, and was also potent against Enterobacteriaceae, *P. aeruginosa*, and *Streptococcus*.

## Introduction

Multidrug-resistant (MDR) pathogens, especially the ESKAPE pathogens (*Enterococcus faecium*, *Staphylococcus aureus*, *Klebsiella pneumoniae*, *Acinetobacter baumannii*, *Pseudomonas aeruginosa*, and *Enterobacter* species), are the leading cause of nosocomial infections throughout the world, which is usually caused by excessive drug usage or prescription and inappropriate use of antimicrobials. Understanding the mechanisms, developing novel antimicrobial agents, and knowing the latest antimicrobial resistance patterns of bacterial pathogens are crucial to combat these public health challenges (Santajit and Indrawattana, 2016; Sheu et al., 2018; Gajdacs, 2019).

Cefoselis is a member of the fourth-generation cephalosporins which exhibit a wider antibacterial spectrum activity than the third-generation cephalosporins to both Gram-negative and Gram-positive bacteria (King et al., 1995). The wide antibacterial spectrum of cefoselis is attributed to the resistance to hydrolysis by the chromosomal β-lactamases and the rapid penetration through the bacterial cell wall (Giamarellos-Bourboulis et al., 2000). However, few reports have been published in China on the antimicrobial activity of cefoselis against common bacterial pathogens. The objective of this study was to better understand the *in vitro* activity of cefoselis against common Gram-positive and Gram-negative bacterial pathogens in China.

## Materials and Methods

### Ethics

The protocol was approved by the Human Research Ethics Committee of Peking Union Medical College Hospital (no. S-K262). Peking Union Medical College Hospital did not require written informed consent from participants because this was an *in vitro* study on bacteria isolates without any private data of the human participants.

### Clinical Isolates

A total of 1188 bacterial isolates derived from 18 hospitals in China (January 2014–December 2016) were studied. The bacterial species distribution is shown in Table 1. The isolates from each of the participating hospitals were re-identified by matrix-assisted laser desorption/ionization time-of-flight mass spectrometry (MALDI-TOF MS) (Bruker Daltonics GmbH, Bremen, Germany) at the Central Lab, Peking Union Medical College Hospital (Beijing), China.

**TABLE 1:** Distribution of bacterial species.

**Organisms**	**Number**	**Percentage**
*E. coli* (ESBL+)	134	11.3
*E. coli* (ESBL−)	107	9.0
*K. pneumoniae* (ESBL+)	118	9.9
*K. pneumoniae* (ESBL−)	106	8.9
*P. mirabilis* (ESBL+)	33	2.8
*P. mirabilis* (ESBL−)	33	2.8
*C. freundii*	30	2.5
*E. aerogenes*	30	2.5
*E. cloacae*	30	2.5
*S. marcescens*	30	2.5
*P. vulgaris*	30	2.5
*A. baumannii*	198	16.7
*P. aeruginosa*	30	2.5
MRSA	97	8.2
MSSA	100	8.4
PSSP	25	2.1
PRSP	15	1.3
Beta-hemolytic *streptococci*	27	2.3
Viridans group *streptococci*	15	1.3
Total	1,188	100.0

*ESBL, extended-spectrum β-lactamase; MRSA, methicillin-resistant *S. aureus*; MSSA, methicillin-sensitive *S. aureus*; PSSP, penicillin-susceptible *S. pneumoniae*; PRSP, penicillin-resistant *S. pneumoniae*.*

### Antimicrobial Susceptibility Test Method

Minimum inhibitory concentrations (MICs) were determined by the broth microdilution method following Clinical and Laboratory Standards Institute (CLSI) guidelines. Thirty-two antimicrobial agents were tested against the isolates, among which 17 agents were against Gram-negative organisms, 24 agents against *Staphylococcus* spp., and 19 agents against *Streptococcus* spp. Cefoselis was obtained from Hansoh Pharma, and the other agents were provided by AstraZeneca. Interpretation of the antimicrobial testing results was based on CLSI M100-S28 (CLSI, 2018). *Escherichia coli* ATCC 25922, *P. aeruginosa* ATCC 27853, *K. pneumoniae* ATCC 700603, *S. aureus* ATCC 29213, and *Streptococcus pneumoniae* ATCC 49619 were used as the quality control strains.

### Extended-Spectrum β-Lactamase Detection

Phenotypic identification of extended-spectrum β-lactamase (ESBL) production in *E. coli*, *K. pneumoniae*, and *Proteus mirabilis*, was carried out using CLSI-recommended methods. If the cefotaxime or ceftazidime MICs were ≥ 2 μg/ml, the MICs of cefotaxime + clavulanic acid (4 μg/ml) or ceftazidime + clavulanic acid (4 μg/ml) were comparatively determined. ESBL production was defined as an eightfold or greater decrease in MICs for cefotaxime or ceftazidime tested in combination with clavulanic acid compared to their MICs without clavulanic acid.

## Results

### *In vitro* Activity of Antimicrobial Agents Against Enterobacteriaceae

Against ESBL-producing *E. coli*, *K. pneumoniae*, and *P. mirabilis* isolates, cefoselis, cefepime, cefotaxime, and ceftriaxone showed relatively low susceptibility rates, with drug resistance rates of > 87%. Against non-ESBL-producing strains, most antibiotics revealed good activity, of which cefoselis, cefepime, showed > 94% antimicrobial susceptibility rates. For *Citrobacter freundii*, *Enterobacter aerogenes*, *Enterobacter cloacae*, *Serratia marcescens*, and *Proteus vulgaris* isolates, the susceptibility rates for cefoselis ranged from 56.7% to 83.3%, which were slightly lower than that of cefepime, with susceptibility rates ranging from 70% to 100%. Meropenem and amikacin exhibited high activity against all the Enterobacteriaceae strains (Tables 2, 3).

**TABLE 2:** *In vitro* activity of antimicrobial agents against ESBL-positive and ESBL-negative *Escherichia coli*, *Klebsiella pneumoniae* and *Proteus mirabilis* strains.

**Antimicrobial**	***E. coli***		***E. coli***		***K. pneumoniae***		***K. pneumoniae***		***P. mirabilis***		***P. mirabilis***	
**agents**	** (ESBL+) (134)**		** (ESBL−) (107)**		**(ESBL+) (118)**		** (ESBL−) (106)**		** (ESBL+) (33)**		** (ESBL−) (33)**	
						
	**%R**	**%S**	**%R**	**%S**	**%R**	**%S**	**%R**	**%S**	**%R**	**%S**	**%R**	**%S**
Piperacillin/tazobactam	12.7	84.3	1.9	97.2	23.7	65.3	2.8	97.2	3.0	93.9	3.0	93.9
Ceftazidime	55.2	30.6	0	100.0	59.3	30.5	1.9	97.2	9.1	90.9	0	100.0
Ceftriaxone	99.3	0.7	0	99.1	94.9	4.2	2.8	94.3	100.0	0.0	0	100.0
Cefotaxime	99.3	0.7	0	100.0	94.9	5.1	3.8	95.3	97.0	0.0	0	100.0
Cefoselis	97.8	2.2	0	100.0	93.2	6.8	0.9	94.3	97.0	3.0	3.0	97.0
Cefepime	91.0	3.7	0	100.0	90.7	6.8	0	100.0	87.9	3.0	0	97.0
Cefoxitin	20.9	58.2	1.9	93.5	18.6	78.0	12.3	84.0	3.0	93.9	0	100.0
Aztreonam	83.6	8.2	0	100.0	81.4	14.4	0.9	99.1	41.4	58.6	21.2	78.8
Ertapenem	0	92.5	0	100.0	0	92.4	0	100.0	0	97.0	0	100.0
Imipenem	0	97.8	0	100.0	0	94.9	0	96.2	15.2	27.3	9.1	45.5
Meropenem	0	100.0	0	100.0	0	100.0	0	100.0	0	100.0	0	100.0
Amikacin	3.0	95.5	0	100.0	7.6	91.5	0.9	99.1	6.1	90.9	0	100.0
Ciprofloxacin	78.4	20.1	29.0	69.2	55.9	40.7	11.3	87.7	90.9	9.1	48.5	48.5
Levofloxacin	72.4	20.9	27.1	71.0	46.6	47.5	10.4	87.7	69.7	27.3	27.3	63.6
Minocycline	47.0	40.3	28.0	54.2	44.1	38.1	21.7	69.8	–	–	–	–
Tetracycline	87.3	12.7	84.1	15.0	66.1	32.2	25.5	67.9	–	–	–	–
Tigecycline	0.7	92.5	0	98.1	3.4	82.2	0.9	90.6	–	–	–	–

*ESBL, extended-spectrum β-lactamase; R, resistant; S, sensitive.*

**TABLE 3:** *In vitro* activity of antimicrobial agents against Enterobacteriaceae strains.

**Antimicrobial agents**	***C. freundii* (30)**		***E. aerogenes* (30)**		***E. cloacae* (30)**		***S. marcescens* (30)**		***P. vulgaris* (30)**	
						
	**%R**	**%S**	**%R**	**%S**	**%R**	**%S**	**%R**	**%S**	**%R**	**%S**
Piperacillin/tazobactam	20.0	73.3	20.0	63.3	26.7	60.0	13.3	83.3	0	93.3
Ceftazidime	30.0	63.3	33.3	60.0	43.3	46.7	3.3	93.3	0	100.0
Ceftriaxone	50.0	43.3	40.0	53.3	60.0	36.7	23.3	73.3	53.3	3.3
Cefotaxime	50.0	43.3	50.0	43.3	60.0	33.3	30.0	60.0	46.7	16.7
Cefoselis	30.0	56.7	10.0	83.3	33.3	56.7	20.0	80.0	3.3	83.3
Cefepime	26.7	70.0	10.0	90.0	13.3	70.0	16.7	80.0	0	100.0
Cefoxitin	63.3	23.3	90.0	3.3	93.3	3.3	80.0	0.0	13.3	73.3
Aztreonam	43.3	56.7	36.7	63.3	56.7	43.3	13.3	86.7	0	96.7
Ertapenem	6.7	90.0	10.0	90.0	16.7	60.0	13.3	86.7	6.7	93.4
Imipenem	6.7	86.7	6.7	50.0	6.7	83.3	16.7	56.7	66.7	6.7
Meropenem	6.7	93.3	3.3	96.7	6.7	93.3	13.3	83.3	3.3	96.7
Amikacin	3.3	90.0	0	100.0	0	100.0	3.3	93.3	3.3	96.7
Ciprofloxacin	26.7	70.0	10.0	83.3	40.0	53.3	23.3	73.3	30.0	66.7
Levofloxacin	26.7	66.7	6.7	90.0	30.0	60.0	13.3	73.3	6.7	80.0
Minocycline	16.7	70.0	10.0	66.7	30.0	63.3	6.7	86.7	–	–
Tetracycline	36.7	60.0	43.3	56.7	36.7	63.3	60.0	10.0	–	–
Tigecycline	0	100.0	0	96.7	0	93.3	0	93.3	–	–

*R, resistant; S, sensitive.*

### *In vitro* Activity of Antimicrobial Agents Against Non-fermentative Gram-Negative Organisms

The most active agents against *A. baumannii* were tigecycline and minocycline, with susceptibility rates of 58.6% and 45.5%, respectively. The other analyzed agents were less effective, with susceptibility rates of < 30%. Furthermore, against *P. aeruginosa* isolates, amikacin exhibited the highest *in vitro* activity, with a susceptibility rate of 93.3%. The susceptibility rates for cefoselis, cefepime, and ceftazidime were all 73.3% each for this organism (Table 4).

**TABLE 4:** *In vitro* activity of antimicrobial agents against *Acinetobacter baumannii* and *Pseudomonas aeruginosa* strains.

**Antimicrobial agents**	***A. baumannii* (198)**		***P. aeruginosa* (30)**	
		
	**%R**	**%S**	**%R**	**%S**
Piperacillin/tazobactam	75.3	23.2	13.3	80.0
Ceftazidime	76.8	20.7	20.0	73.3
Ceftriaxone	78.8	7.1	96.7	0
Cefotaxime	78.8	14.6	100.0	0
Cefoselis	80.8	18.7	26.7	73.3
Cefepime	75.8	19.7	20.0	73.3
Cefoxitin	96.5	2.5	96.7	0
Aztreonam	84.3	5.1	20	73.3
Ertapenem	–	–	–	–
Imipenem	75.3	24.7	20.0	80.0
Meropenem	75.3	24.2	10.0	76.7
Amikacin	69.7	29.8	6.7	93.3
Ciprofloxacin	76.8	21.2	16.7	76.7
Levofloxacin	59.6	23.2	16.7	76.7
Minocycline	28.3	45.5	–	–
Tetracycline	81.3	16.2	–	–
Tigecycline	22.7	58.6	96.7	3.3

*R, resistant; S, sensitive.*

### *In vitro* Activity of Antimicrobial Agents Against MRSA and MSSA

Against methicillin-resistant *S. aureus* (MRSA) strains, linezolid, vancomycin, and teicoplanin exhibited a susceptibility rate of 100% each, followed by tigecycline (97.9%), and trimethoprim–sulfamethoxazole (TMP-SMX) (94.8%). All strains were resistant to ceftazidime, ceftriaxone, cefoselis, and cefepime. Against 100 methicillin-sensitive *S. aureus* (MSSA) strains, most antibiotics showed good activity, except for ampicillin, tetracycline, and erythromycin. All strains were susceptible to ceftazidime, ceftriaxone, cefoselis, and cefepime (Table 5).

**TABLE 5:** *In vitro* activity of antimicrobial agents against MRSA and MSSA strains.

**Antimicrobial agents**	**MRSA (97)**		**MSSA (100)**	
	**%R**	**%S**	**%R**	**%S**
Ampicillin	100.0	0	90.0	10
Oxacillin	100.0	0	0	100.0
Amoxicillin/clavulanate	100.0	0	0	100.0
Piperacillin/tazobactam	100.0	0	0	100.0
Ceftaroline	4.1	35.1	0	95.0
Ceftazidime	100.0	0	0	100.0
Ceftriaxone	100.0	0	0	100.0
Cefoselis	100.0	0	0	100.0
Cefepime	100.0	0	0	100.0
Doripenem	100.0	0	0	100.0
Meropenem	100.0	0	0	100.0
Gentamicin	49.5	38.1	18.0	81.0
Levofloxacin	79.4	20.6	23.0	75.0
Moxifloxacin	77.3	19.6	15.0	77.0
Trimethoprim–sulfamethoxazole	5.2	94.8	2.0	98.0
Clindamycin	47.4	50.5	21.0	78.0
Daptomycin	0	99	0	99.0
Erythromycin	82.5	8.2	38.0	60.0
Linezolid	0	100.0	0	100.0
Vancomycin	0	100.0	0	100.0
Teicoplanin	0	100.0	0	100.0
Minocycline	0	88.7	0	100.0
Tetracycline	60.8	36.1	43.0	52.0
Tigecycline	2.1	97.9	0	100.0

*MRSA, methicillin-resistant *S. aureus*; MSSA, methicillin-sensitive *S. aureus*; R, resistant; S, sensitive.*

### *In vitro* Activity of Antimicrobial Agents Against *Streptococcus* Strains

For the penicillin-susceptible *S. pneumoniae* (PSSP), beta-hemolytic *Streptococcus* strains, and viridans group *Streptococcus* strains, cefoselis and cefepime both showed very high antimicrobial activities, with susceptibility rates of > 90%. Against 15 penicillin-resistant *S. pneumoniae* (PRSP) strains, the susceptibility rate of cefoselis was higher than that of cefepime (60.0% *vs*. 40.0%). Linezolid, vancomycin, and tigecycline exhibited 100% antimicrobial activity against all the *Streptococcus* strains (Table 6).

**TABLE 6:** *In vitro* activity of antimicrobial agents against *Streptococcus* strains.

**Antimicrobial agents**	**PSSP (25)**		**PRSP (15)**		**Beta-hemolytic**		**Viridans group**	
					***streptococci* (27)**		***streptococci* (15)**	
				
	**%R**	**%S**	**%R**	**%S**	**%R**	**%S**	**%R**	**%S**
Penicillin	0	100.0	100.0	0	0	100.0	0	100.0
Amoxicillin/clavulanate	0	100.0	40.0	40.0	–	–	–	–
Ceftaroline	0	100.0	0	100.0	0	92.6	0	100.0
Ceftazidime	4.0	92.0	86.7	0	7.4	92.6	13.3	86.7
Ceftriaxone	0	96.0	40.0	53.3	0	92.6	6.7	91.7
Cefoselis	4.0	96.0	0	60.0	7.4	92.6	0	100.0
Cefepime	4.0	96.0	20.0	40.0	7.4	92.6	6.7	93.3
Doripenem	0	100.0	0	100.0	0	92.6	0	100.0
Meropenem	4.0	92.0	40.0	6.7	0	92.6	0	100.0
Levofloxacin	4.0	96.0	0	100.0	46.2	53.8	6.7	80.0
Moxifloxacin	32.0	68.0	0	100.0	–	–	–	–
Clindamycin	100.0	0	100.0	0	74.1	22.2	66.7	33.3
Daptomycin	–	–	–	–	–	92.7	–	93.3
Erythromycin	76.0	24.0	100.0	0	48.1	51.9	80.0	13.3
Linezolid	0	100.0	0	100.0	0	100.0	0	100.0
Vancomycin	0	100.0	0	100.0	0	100.0	0	100.0
Minocycline	12.0	48.0	6.7	73.3	33.3	48.1	6.7	80.0
Tetracycline	88.0	8.0	93.3	6.7	74.1	22.2	66.7	33.3
Tigecycline	0	100.0	0	100.0	0	100.0	0	100.0

*PSSP, penicillin-susceptible *S. pneumoniae*; PRSP, penicillin-resistant *S. pneumoniae*; R, resistant; S, sensitive.*

### Comparison of Cefoselis and Cefepime Against Common Clinical Pathogens

Cefoselis exhibited a slightly lower antimicrobial activity than cefepime against Enterobacteriaceae and non-fermentative Gram-negative organisms, but a little higher activity than cefepime against MRSA, MSSA, PSSP, beta-hemolytic *Streptococcus*, and viridans group *Streptococcus* strains. The cumulative percentage MIC distributions of cefoselis and cefepime against common clinical pathogens are shown in Table 7.

**TABLE 7:** Cumulative percentage MIC distributions of cefoselis and cefepime against common clinical pathogens collected in China.

**Species (*n*) and drug**		**Cumulative% isolates at or below various MICs (μg/ml)^a^**															
		
		** ≤ 0.015**	**0.03**	**0.06**	**0.12**	**0.25**	**0.5**	**1**	**2**	**4**	**8**	**16**	**32**	**64**	**128**	**256**	** > 256**
*E. coli* (ESBL+) (134)	Cefoselis			1.5	2.2	2.2	2.2	2.2	2.2	2.2	2.2	3.7	8.2	11.9	20.1	20.9	**100**
	Cefepime			0.7	1.5	2.2	2.2	2.2	3.7	5.2	9.0	18.7	26.9	32.8	36.6	40.3	**100**
*E. coli* (ESBL-) (107)	Cefoselis	1.9	17.8	71.0	**92.5**	98.1	98.1	98.1	100								
	Cefepime	0.9	16.8	66.4	**94.4**	97.2	100										
*K. pneumoniae* (ESBL+) (118)	Cefoselis		0.8	4.2	5.1	5.9	5.9	6.8	6.8	6.8	6.8	6.8	6.8	12.7	16.1	16.9	**100**
	Cefepime		0.8	4.2	5.1	5.1	5.1	6.8	6.8	6.8	9.3	11.9	17.8	21.2	26.3	31.4	**100**
*K. pneumoniae* (ESBL-) (106)	Cefoselis	1.9	30.2	60.4	81.1	**91.5**	93.4	93.4	94.3	97.2	99.1	99.1	100				
	Cefepime	3.8	46.2	67.9	82.1	**91.5**	92.5	96.2	100								
*P. mirabilis* (ESBL+) (33)	Cefoselis							3.0	3.0	3.0	3.0	3.0	6.1	9.1	9.1	9.1	**100**
	Cefepime					3.0	3.0	3.0	3.0	3.0	12.1	12.1	21.2	30.3	30.3	30.3	**100**
*P. mirabilis* (ESBL-) (33)	Cefoselis	3.0	39.4	48.5	60.6	66.7	75.8	81.8	**97.0**	97.0	97.0	100					
	Cefepime		42.4	45.5	51.5	57.6	69.7	87.9	**97.0**	97.0	100						
*C. freundii* (30)	Cefoselis	26.7	43.3	46.7	50.0	53.3	53.3	56.7	56.7	60.0	70.0	70.0	73.3	80.0	83.3	83.3	**100**
	Cefepime	30.0	46.7	53.3	56.7	60.0	63.3	63.3	70.0	73.3	73.3	83.3	83.3	83.3	**90.0**	93.3	100
*E. aerogenes* (30)	Cefoselis	10.0	33.3	43.3	50.0	63.3	70.0	73.3	83.3	**90.0**	90.0	90.0	90.0	90.0	90.0	93.3	100
	Cefepime	6.7	43.3	46.7	63.3	76.7	86.7	**90.0**	90.0	90.0	90.0	93.3	96.7	96.7	96.7	96.7	100
*E. cloacae* (30)	Cefoselis	3.3	30.0	36.7	36.7	43.3	46.7	46.7	56.7	60.0	66.7	66.7	83.3	86.7	**93.3**	93.3	100
	Cefepime	10.0	30.0	33.3	43.3	50.0	56.7	63.3	70.0	76.7	86.7	**93.3**	96.7	96.7	96.7	100	
*S. marcescens* (30)	Cefoselis	30.0	70.0	73.3	76.7	76.7	76.7	76.7	80.0	80.0	80.0	80.0	80.0	80.0	80.0	80.0	**100**
	Cefepime	10.0	66.7	73.3	76.7	76.7	76.7	76.7	80.0	80.0	83.3	86.7	86.7	86.7	86.7	86.7	**100**
*P. vulgaris* (30)	Cefoselis		3.3	30.0	46.7	60.0	70.0	76.7	83.3	**93.3**	96.7	96.7	96.7	96.7	96.7	96.7	100
	Cefepime		10.0	36.7	50.0	76.7	86.7	**100**									
*A. baumannii* (198)	Cefoselis	1.5	2.5	3.5	5.1	6.6	8.6	16.2	18.7	18.7	18.7	19.2	21.2	26.3	40.4	71.2	**100**
	Cefepime	1.0	2.0	3.5	4.5	5.6	6.6	12.1	16.2	18.7	19.7	24.2	36.9	52.0	84.3	**93.9**	100
*P. aeruginosa* (30)	Cefoselis						3.3	36.7	50.0	63.3	73.3	73.3	73.3	76.7	**96.7**	96.7	100
	Cefepime						3.3	53.3	56.7	70.0	73.3	80.0	80.0	**96.7**	100		
MRSA (97)	Cefoselis							2.1	3.1	18.6	27.8	32.0	**96.9**	100			
	Cefepime								1.0	2.1	5.2	13.4	18.6	25.8	28.9	28.9	**100**
MSSA (100)	Cefoselis	3.0	3.0	3.0	3.0	3.0	4.0	64.0	82.0	87.0	**90.0**	92.0	100				
	Cefepime	3.0	3.0	3.0	3.0	3.0	3.0	5.0	57.0	78.0	83.0	87.0	88.0	89.0	**90.0**	93.0	100
PSSP (25)	Cefoselis	32.0	72.0	80.0	84.0	**92.0**	92.0	96.0	96.0	96.0	96.0	96.0	100				
	Cefepime	4.0	72.0	76.0	80.0	88.0	**92.0**	96.0	96.0	96.0	96.0	96.0	100				
PRSP (15)	Cefoselis						6.7	40.0	40.0	86.7	**100**						
	Cefepime							20.0	60.0	**100**							
Beta-hemolytic *streptococci* (27)	Cefoselis	40.7	85.2	88.9	**92.6**	92.6	92.6	92.6	92.6	92.6	92.6	92.6	92.6	92.6	92.6	92.6	100
	Cefepime	37.0	40.7	85.2	**92.6**	92.6	92.6	92.6	92.6	92.6	92.6	92.6	92.6	92.6	92.6	92.6	100
Viridans group *streptococci* (15)	Cefoselis	26.7	46.7	93.3	**100**												
	Cefepime	26.7	33.3	40.0	53.3	**93.3**	93.3	93.3	93.3	93.3	100						

*ESBL, extended-spectrum β-lactamase; MRSA, methicillin-resistant *S. aureus*; MSSA, methicillin-sensitive *S. aureus*; PSSP, penicillin-susceptible *S. pneumoniae*; PRSP, penicillin-resistant *S. pneumoniae*; MIC, minimum inhibitory concentration. ^*a*^MIC90 values are in boldface.*

## Discussion

The Enterobacteriaceae family is a major group of pathogens causing several community- and hospital-acquired infections, among which the ESBL rates in *E. coli* and *K. pneumoniae* in China have been reported as high as 60–70% and 30–40%, respectively (Yang et al., 2010, 2013). According to our previous study, the genotype distribution of ESBL-producing strains among bacterial species was diverse, and *blaCTX-M* was the major ESBL gene, with occurrences in 99.5% of *E. coli*, 91.1% of *K. pneumoniae*, and 97.5% of *P. mirabilis* strains (Yang et al., 2015). In the present study, cefoselis and cefepime both showed poor activities against ESBL-producing *E. coli*, *K. pneumoniae*, and *P. mirabilis*, which may be attributed to the specific ESBL genes present in China, albeit further studies are needed for confirmation.

Against other Enterobacteriaceae strains, cefoselis exhibited a slightly lower antimicrobial activity than cefepime, but a higher activity than third-generation cephalosporins. The relatively high activities of fourth-generation cephalosporins against Enterobacteriaceae may be attributed to the low affinity for chromosome-mediated AmpC β-lactamases (D’Angelo et al., 2016), which are the common β-lactamases in Enterobacteriaceae isolates from China. A multicenter, double-blind, randomized clinical trial in China revealed equal clinical efficacy and safety of intravenous cefoselis and cefepime injection for the treatment of acute, moderate, and severe bacterial infections (Liu et al., 2014).

*Acinetobacter baumannii* was one of the bacteria considered to be of maximum resistance and is classified as a priority category according to the bacterial groups classified by priority categories of need for new antibiotics (Tacconelli and Magrini, 2017). In this study, *A. baumannii* exhibited low susceptibility to most of the tested antibiotics, with susceptibility rates ranging from 5.1 to 58.6%. Against *P. aeruginosa*, cefoselis and cefepime showed equal antimicrobial activities for this organism, with susceptibility rates of 73.3% each, which are slightly higher than those in a previous study in China; thus, these two antibiotics could be used to treat infections caused by *P. aeruginosa*, in combination with other antibiotics (Zhang et al., 2016).

Although the prevalence of MRSA in China showed a markedly decreasing trend from 69.0% in 2005 to 35.3% in 2017, as per the China Antimicrobial Surveillance Network (CHINET) program, MRSA remains a major pathogen responsible for nosocomial infections (Hu et al., 2018). No isolates were found to be resistant to vancomycin, linezolid, and teicoplanin in this study. Tigecycline and TMP-SMX also showed good activities, which were similar to previous studies (Zhao et al., 2012; Zhang et al., 2015). Vancomycin, linezolid, and TMP-SMX were recommended by the Infectious Diseases Society of America (IDSA) to treat MRSA infections (Liu et al., 2011).

Teicoplanin can be an effective alternative to vancomycin for treating patients infected by MRSA as the two therapies are similar in both efficacy and safety (Peng et al., 2013). Tigecycline was often recommended as a second- or third-line agent for MRSA infections when alternative agents cannot be used (Rodvold and McConeghy, 2014). Ceftaroline fosamil was the first FDA-approved cephalosporin with any activity against MRSA, but the low susceptibility among MRSA isolates in China needs attention (Lodise and Low, 2012; Zhang et al., 2015).

The bacterial isolates were collected from 2014 to 2016, and the susceptibility has certainly changed in the last 5 years for most organisms. More recently collected strains should be involved in further studies. This is a limitation of the study. In conclusion, cefoselis exhibited good antimicrobial activity against non-ESBL[Frame1]-producing *E. coli*, *K. pneumoniae*, *P. mirabilis*, and MSSA and was also potent against other Enterobacteriaceae, *P. aeruginosa*, and *Streptococcus*.

## Data Availability Statement

The datasets generated for this study are available on request to the corresponding author.

## Author Contributions

J-WC, J-RS, and TK wrote the manuscript. S-YY, GZ, J-JZ, YY, and S-MD collaborated the strains and performed the antimicrobial susceptibility tests. MX, Q-WY, and Y-CX designed and supervised the study.

## Conflict of Interest

The authors declare that the research was conducted in the absence of any commercial or financial relationships that could be construed as a potential conflict of interest.
